# Anisotropic tough poly(vinyl alcohol)/graphene oxide nanocomposite hydrogels for potential biomedical applications

**DOI:** 10.1039/c8ra00340h

**Published:** 2018-04-10

**Authors:** Qiaomei Luo, Yangyang Shan, Xia Zuo, Jiaqi Liu

**Affiliations:** Department of Chemistry, Capital Normal University Beijing 100048 P. R. China liujq@cnu.edu.cn +86 10 68903040 +86 10 68902974

## Abstract

Hydrogels, one of the most important bioinspired materials, are receiving increasing attention because of their potential applications as scaffolds for artificial tissue engineering and vehicles for drug delivery, *etc.* However, these applications are always severely limited by their microstructure and mechanical behavior. Here we report the fabrication of a tough polyvinyl alcohol/graphene oxide (PVA/GO) nanocomposite hydrogel through a simple and effective directional freezing–thawing (DFT) technique. The resulting hydrogels show well-developed anisotropic microstructure and excellent mechanical properties with the assistance of DFT method and lamellar graphene. The hydrogels with anisotropic porous structures that consisted of micro-sized fibers and lamellas exhibit high tensile strengths, up to 1.85 MPa with a water content of 90%. More interestingly, the PVA/GO composite hydrogels exhibit the better thermostability, which can maintain the original shape when swollen in hot water (65 °C). In addition, the hydrogels with biocompatibility show good drug release efficiency due to the unique hierarchical structure. The successful synthesis of such hydrogel materials might pave the way to explore applications in biomedical and soft robotics fields.

## Abstract 2

Tough PVA/GO nanocomposite hydrogel with well-developed anisotropic microstructure and excellent mechanical properties.

## Graphical abstract

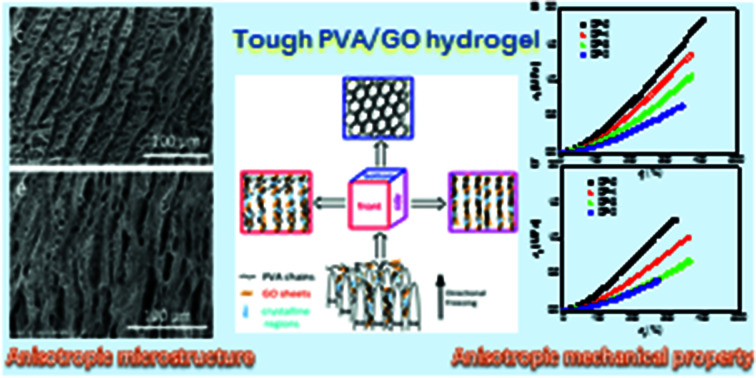

## Introduction

Hydrogels, defined as a unique soft material with 3D gel networks, have a number of applications in the biomedical and industrial fields.^1–3^ Compared with many biological gels with well-developed microstructures, the normal synthetic hydrogels and their lower mechanical strength, due to the structural inhomogeneity in their network,^4^ further impede their further practical utilization. In recent years, many efforts have been focused on enhancing the mechanical properties of hydrogels. Five typical tough hydrogels, topological (TP) gel or slide-ring (SR) gel,^5^ nanocomposite (NC) gel,^6^ double network (DN) gel,^7^ tetra-arm polyethylene glycol (PEG) based gel,^8^ and macromolecular microsphere composite (MMC) gel,^9,10^ have been developed successively. But only a few hydrogels based on the DN and MMC gels with water content of 70–85% exhibit high mechanical strength,^2,9^ and most of the resulting hydrogels are isotropic in both microstructure and properties.

Poly(vinyl alcohol) (PVA) hydrogels have drawn greater attention in biomedical fields because of their non-toxicity, biocompatibility and good mechanical properties. And most of PVA hydrogels can be prepared by traditional repeated freezing and thawing (FT) method.^11^ Directional freezing–thawing technique (DFT) can possibly developed PVA hydrogels with anisotropic mechanical properties.^12–14^ But the conventional hydrogels in practical applications are also limited by their isotropic microstructure and poor mechanical properties, especially in much higher water content.

In the past ten years, many efforts have been made to fabricate graphene or graphene oxide (GO) composite hydrogels with excellent mechanical properties due to its extraordinary electronic, thermal, and mechanical properties.^15–17^ For instance, Shi *et al.* have reported a new composite hydrogel with GO and PVA,^18^ which depends on the assembly of GO sheets and the cross-linking effect of PVA chains *via* hydrogen bonding as the dominant force but did not demonstrate its capability strength. Huang and co-workers prepared a GO/PVA composite hydrogel with water content of 82% by a freeze/thaw method, improving the mechanical properties (132% increasing in tensile strength) with the addition of 0.8 wt% of GO.^19^ In this case, the GO nanosheets also act as physical crosslinked agent by hydrogen bonds. However, the hydrogels do not show anisotropic in both microstructure and properties. And the well-defined hierarchical design of tough hydrogels with high water content has not been achieved. Our group reports a jellyfish-like material employing PVA/GO hydrogel,^18,20^ the biomimetic gel exhibits excellent mechanical properties (0.17 MPa in tensile strength) with super-high water of 97%. Based on the mentioned above, we thus surmised that the tough hydrogels could be obtained if graphene sheets were incorporated into the polymer matrix orderly with assistance of DFT method and excellent thermal property of graphene sheets.

In the present work, we first attempted to synthesize the PVA/GO composite hydrogel with micro-structure and macro-properties by DFT technique. The resulting hydrogels with high water content of 90% show excellent tensile properties. More importantly, the PVA/GO gels show aligned hierarchical microstructure and anisotropic mechanical properties with assistance of DFT method and lamellar graphene. In addition, the applications of drug released and biocompatibility were investigated.

## Experimental

### Materials and methods

PVA was supplied by the Sinopharm Chemical Reagent Co, Ltd. (Shanghai, China). Graphene oxide was synthesized from the modified Hummers' method,^19,30^ and the specific preparation process was reported in our previous work. The GO aqueous solutions with different concentrations were obtained by dispersing GO in deionized water by ultrasonication and mechanically stirring at room temperature (RT) for 2 hours until the mixture remained continuous flocculent and without significant solid material when shaking gently. Reagents for fluorescence and breeding of HeLa cells were also commercially available. All other reagents were analytical reagent grade and purchased from Beijing Chemical Reagent Company (Beijing, China) without further purification. Purified water was used throughout all the experiments.

### Synthesis of hydrogels

The anisotropic PVA/GO composite hydrogels with high mechanical strength were prepared with the directional freezing–thawing (DFT) method. Specifically, 10 g of the PVA was accurately weighed and swollen into 90 mL of deionized water for 12 h at RT, and then the mixture was transferred to a 200 mL three-necked flask and heated to 90 °C for 4–5 hours under mechanical agitation until PVA solution was completely dissolved. Then the PVA solution was diluted to 10% with different concentrations of GO aqueous solution under the stirring evenly and ultrasonic treatment after the dissolved solution cooled nearly to RT, naturally, concentrations of well-dispersed PVA/GO mixed solutions were obtained. Eventually, the PVA/GO colloidal dispersions were transferred into molds and immersed into directional refrigeration device with suitable working speed till the ice crystal grew to the upper edge of the mold, after then, a directional freezing experiment was finished. After placed at RT for 2 h, the next freeze could be carried out. In this work, the freezing number was designed to 2–5 times.

For comparison, PVA hydrogels without GO were also obtained by using the same DFT method, in addition, the normal PVA/GO hydrogels were prepared with traditional repeated freezing and thawing (FT) method, the details were that freezing PVA/GO mixture at −20 °C for 12 h, and then thawed it at 25 °C for 2 h. Finally, the steps were repeated for several times to obtain the FT PVA/GO hydrogel samples.

### Characterization

#### Scanning electron microscopy (SEM) investigation

The samples cut from the as prepared PVA/GO hydrogels were immersed into liquid nitrogen for about 30 min and then subsequently freeze dried in a FD-1B-50 vacuum freeze dryer (Beijing Boyikang Laboratory Apparatus Co., Ltd., Beijing, China) for about 48 h. The freeze-dried samples were cut along the directions parallel and perpendicular to the freezing direction, respectively. And then the fresh surfaces were exhibited and sputter coated with Pt for 1 min to observe the microstructure by a scanning electron microscope (Hitachi S-4800, Tokyo, Japan).

#### Mechanical testing

The hydrogel samples were cut into dumbbell shaped specimens from directions parallel and perpendicular to the freezing direction and standardized as DIN-53504 S3 for tensile testing, and an Instron 3366 electronic universal testing machine (Instron Corporation, MA, USA) with 100 N load cells was employed for this test. The specific method including tensile stress (*σ*_t_), tensile strain (*ε*_t_), fracture tensile stress (*σ*_b_), fracture tensile strain (*ε*_b_) and elastic modulus (*E*) were defined and calculated as described in our previous work.^10^ During the mechanical measurements, at least three specimens were tested to receive reliable data.

#### FTIR characterization

Fourier-transform infrared (FTIR) characterization was performed with a Nicolet-380 FTIR spectrometer (USA) using the KBr method in the range of 400–4000 cm^−1^.

#### Swelling performance

The PVA/GO composite hydrogel samples with water content of 90 wt% were cut into small pieces, then accurately weighed and placed them in the wire mesh in 65 °C water bath. After then gel samples were taken out continuously at regular intervals with absorbing of water droplets on the surface by filter paper. Finally, the quality of gels was weighed exactly until it remained substantially stable without changing. Then the mass change curves of the gel samples were obtained at different swollen times.

#### Biocompatibility examination

Firstly, the gel samples were cut into flakes with appropriate size and put them into the Petri dishes sterilized under the UV lamp irradiation for more than 30 min, after then the samples were removed into the clean bench, meaning the sterilization treatment was completed. Afterwards, the sterilized gel samples were soaked in bovine serum culture medium for 24 h, and observed the system under the microscope to ensure that the culture medium was not contaminated. At last, HeLa cells were adherent incubated on a gel samples and the growing situation of cells was observed by a microscope at regular intervals.

#### Drug release performance

Firstly, 3 mmol L^−1^ of the common vitamin B12 (VB12) used as the drug model was loaded into PVA/GO mixed solution, then the PVA/GO/VB12 composite hydrogels were prepared by DFT. A certain size of the composite gel samples was cut and swollen into 20 mL PBS buffer and HCl solution with pH of 7.4 and 1.7, respectively. Finally, 3.5 mL solution was removed from the swelling system at intervals for UV-Vis spectroscopy. After then the drug release concentration and release rate were calculated according to the absorption curves.

## Results and discussion

**Fig. 1:**
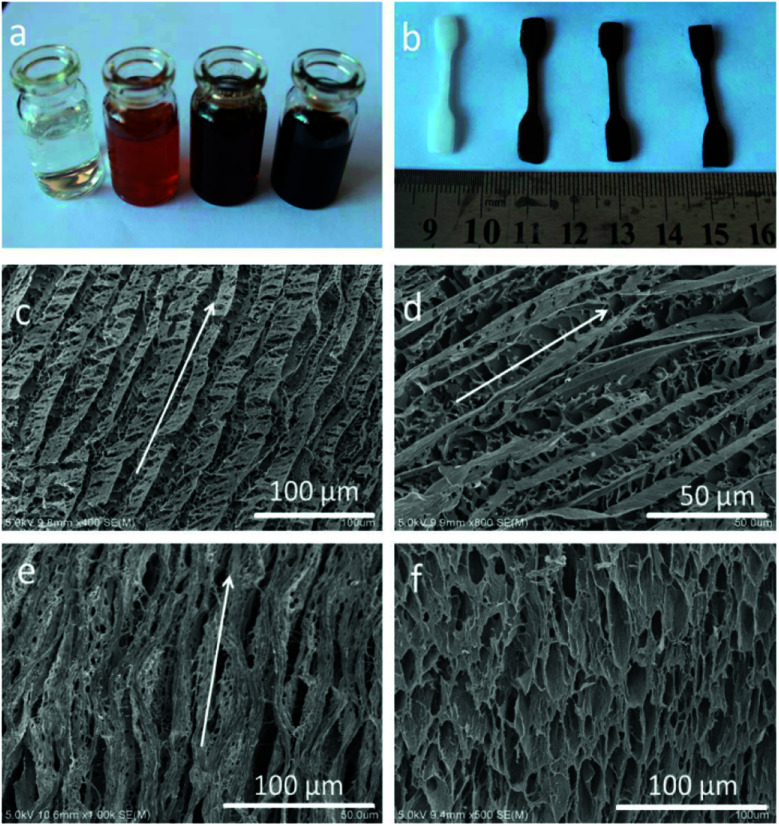
Photographs of the PVA/GO solutions (a), dumbbell-shaped PVA/GO hydrogel specimens (b) with different GO concentrations (GO0, GO2, GO4 and GO8). SEM micrographs of PVA/GO hydrogel prepared with DFT method from the directions front (c and d), side (e) and bottom (f) to the freezing direction indicated with white arrows. (c–f) PVA10/GO8 hydrogel.

We employed a versatile, simple and inexpensive technique to prepare PVA/GO composite hydrogels, namely, Directional freezing–thawing (DFT) method,^14,21^ many water-soluble polymers and composite materials with aligned macroporous structures were successfully prepared by this freezing technique, including aqueous poly(vinyl alcohol) (PVA) solution. In this present work, aligned microchannels in the PVA/GO composite hydrogel have been formed as the growing of ice crystals, and the strong interactions between the PVA chains render its ability to sustain itself after freezing treatment. More importantly, GO sheets with many oxygen-containing functional groups can be well dispersed in PVA matrix due to strong H-bonding between graphene sheets and the PVA chains. The composite PVA/GO with higher water content can be easily achieved owing to the improvement in hydrophilicity with the introduction of GO, which is the strongest material ever measured.^15–17^ Herein, we successfully prepared a fishbone-like hydrogel material based on the directional freezing technique and the unique 2D planar structure of GO. Fig. 1a–f presents the typical macroscopic pictures and scanning electron microscope (SEM) images of PVA/GO composite hydrogels with 90 wt% water content. As shown in Fig. 1a and b, the color of the gels changed from milky to black with the increase of GO concentrations (*C*_GO_) (0, 2, 4, and 8 mg mL^−1^), which was consistent with the color of different concentrations of GO aqueous dispersions. The photo provided direct evidence that the homogeneous exfoliated of GO solutions could be well dispersed in PVA matrix.

Moreover, according to the SEM images of as prepared PVA/GO gel samples shown in Fig. 1c–f, the different directions of PVA/GO gel samples were investigated, which located at the micro-region of hydrogel sample, parallel and perpendicular to the volatilize direction of liquid nitrogen. For example, the freeze-dried samples show anisotropic macroporous structures with aligned microchannels in the parallel direction (Fig. 1c and d). This “fishbone” morphology has been found in our previous work,^14^ which prepared by the directional freezing of pure PVA aqueous solutions. Differently, the resulting PVA/GO gel sample in this case showed a much tight microporous structure, consisting of a series of aligned microchannels parallel to the freezing direction. One major reason was that the high water content of hydrogel sample was employed (90 wt%) during the freezing-dry process, when a large number of ice crystals removed, amount of PVA chains build a 3D micropore structure to form the gel. On the other hand, the large 2D lamellar of graphene have ability to sustain the inner structure of hydrogel to make sure the unique and regular microstructure can be formed.^22^ However, it is difficult to observe the self-standing graphene sheets in the SEM images, mainly attribute to large numbers of PVA chains were dispersed into the polymer matrix to cover or entangle the GO sheets.

**Scheme 1:**
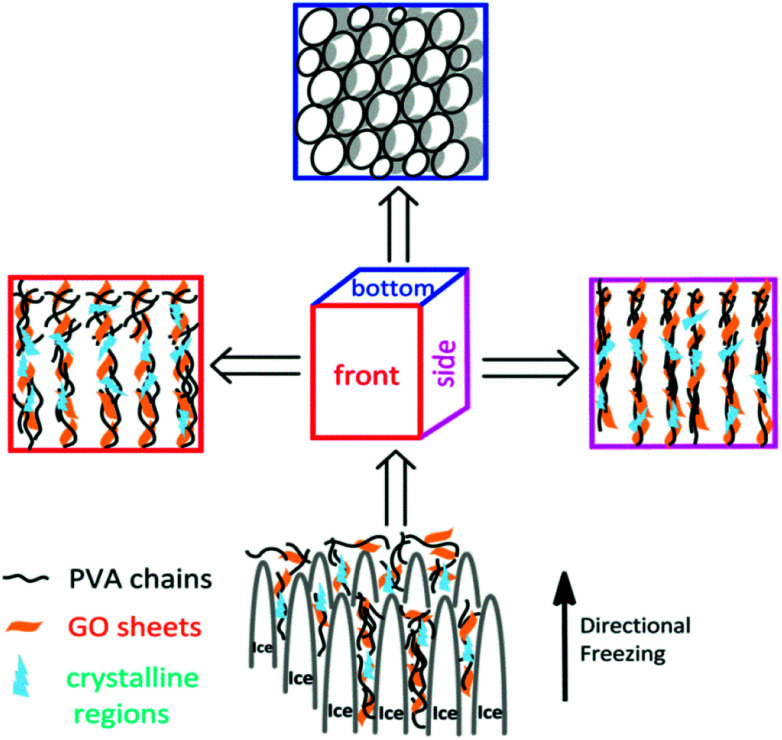
Schematic presentation of the formation of microstructure from different freezing direction.

The aligned lamella microstructure also could be observed in the vertical section along an aligned axis of the PVA/GO gel sample and the SEM micrographs are shown in Fig. 1e. After freezing process, the side surface of gel sample exhibited the well-defined directional structure and the width of the aligned channels could be varied from 5–10 μm. We proposed a feasible mechanism for the formation of aligned lamella structure as follows. In fact, the ice crystals are growing in 3D scale with the volatilize direction of liquid nitrogen during the directional freezing process. As schematically illustrated in Scheme 1, the aligned microstructure could also be formed on the side surface of sample, similar to the front surface. However, due to the smaller cross-sectional area, the proportion of PVA crystalline regions and graphene sheets began to decrease with wider dispersion of phase separation than other side. When the larger ice crystals removed, the highly ordered layer structure was formed under the action of ice crystals.

After that, aligned tubular pores of approximately 10–20 μm in diameter were observed from bottom of the gel sample using SEM (Fig. 1f), which point one direction parallel to the growing of ice crystals, among these microspores, both through- and closed-holes can be observed in the SEM images. When the freezing process completed, the removal of wedge ice template by freeze drying can lead to the formation of tubular pore structures (Scheme 1).

**Fig. 2:**
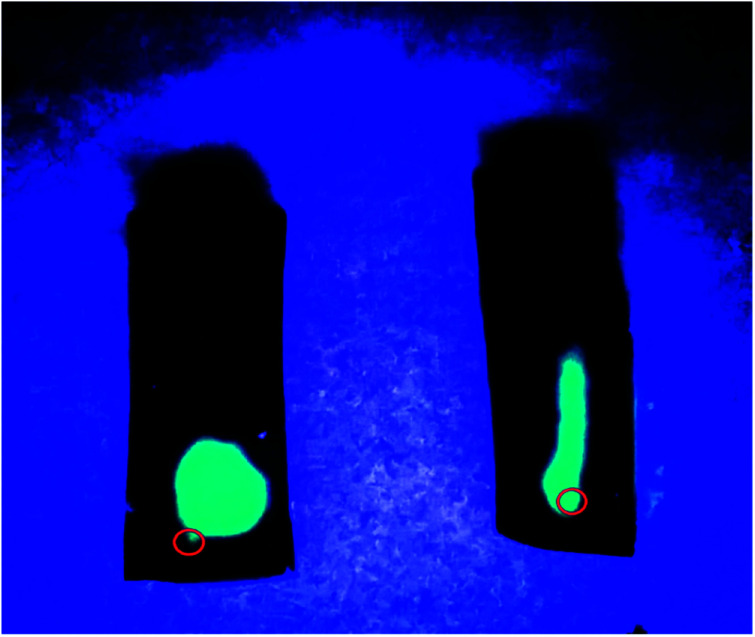
Photographs of two PVA/GO hydrogel specimens with DFT (right) and FT (left) method after injection of luminescent material under UV light. The red circles point the position of injection.

To further confirm these unique anisotropic microstructures, we carried out an interesting test employed two hydrogel samples prepared by different freezing technique (Fig. 2). Specifically, the luminescence solution under UV light was injected slowly into the two samples with a microsyringe along the freezing direction, after then we found that a long and narrow light path along the injection point was showed in the middle of the sample prepared with DFT method, suggesting that the formation of large and long channels after freezing is beneficial to the diffusion of luminescence solution along freezing direction, and another samples showed the random diffusion after injection, which is attributed to the formation of disordered microstructure in the hydrogel through FT treatment.

Besides the microstructure of the resulting hydrogels, we also investigated the mechanical properties of PVA/GO composites hydrogels. The number of DFT cycles and *C*_GO_ are the two important parameters to influence the property of hydrogels. In this work, to ensure the uniform dispersion of GO, the maximum of *C*_GO_ was selected as 8 mg mL^−1^. The water content of the resulting hydrogels was 90%.

**Fig. 3:**
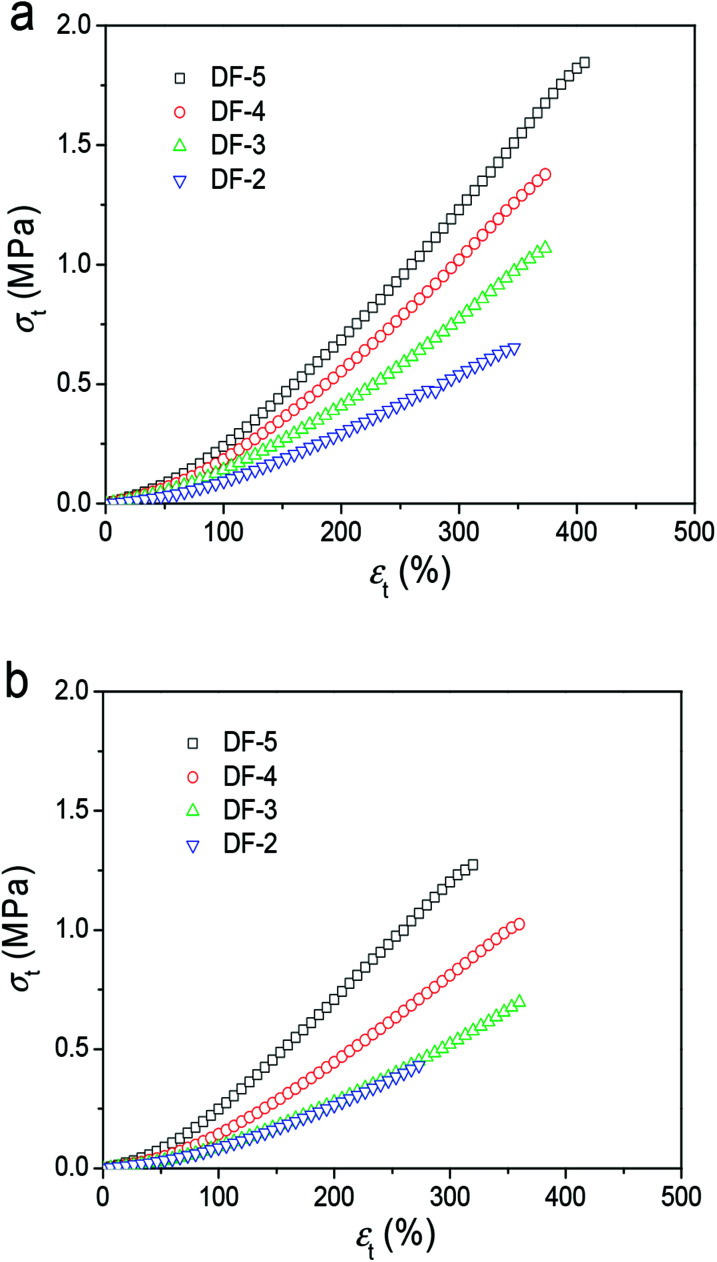
Typical tensile stress–strain curves of PVA/GO8 hydrogels with different DF cycles with direction freezing method (directions parallel (a) and perpendicular (b) to the freezing direction).

Specifically, the tensile stress–strain curves in Fig. 3 summarized the tensile properties of three gel samples parallel and perpendicular to the freezing direction from 2 DFT cycles to 5 DFT cycles. The fracture tensile stress (*σ*_b_) and elastic modulus (*E*) increase significantly with the increasing of DFT cycle. In the parallel direction, the *σ*_b_ increased from 0.60 MPa at 2 DFT cycles to 1.80 MPa at 5 cycles. In the perpendicular direction, the *σ*_b_ decrease to 0.40 MPa after 2 DFT cycles; while 1.30 MPa at 5 DFT cycles. These results mentioned above show that the PVA/GO composite hydrogel exhibit the anisotropic mechanical properties prepared by DFT method.

Furthermore, the mechanical properties of resulting hydrogels with different *C*_GO_ were investigated. As shown in Table 1, the PVA/GO hydrogel had an obvious anisotropy in *σ*_b_ with the increasing of *C*_GO_. In the parallel to the volatilize direction of liquid nitrogen, the breaking strength of gel with 8 mg mL^−1^ GO is up to 1.85 MPa, which is much higher than pure PVA gel without GO (0.55 MPa). More interestingly, the more concentration of GO in the polymer matrix, the bigger strength difference in two freezing direction, which is 0.70 MPa at 8 mg mL^−1^ and 0.15 MPa without GO. The possible reason is that the excellent thermal conductivity of GO sheets can make more PVA crystallites to disperse in parallel directions.^23^ For comparison, the PVA/GO hydrogel formed by TF method was also investigated for its tensile properties. As shown in Table 1, the *σ*_b_ in two directions had no significant difference, indicating the formation of isotropic gels. Furthermore, there are some differences in mechanical properties between the FT and DFT gels. For instance, the *σ*_b_ of DFT PVA/GO4 gel (1.42 MPa) is lower than those of the FT gel (1.45 MPa), while the fracture stress of DFT PVA/GO8 gel is higher than those of FT gel (1.72 MPa).

**Table 1:** The *σ*_b_ of the PVA and PVA/GO composite hydrogels in the parallel (para.) and perpendicular (perp.) directions with DFT and FT method

*C*_PVA_ (wt%)	*C*_GO_ (mg mL^−1^)	*σ*_b_ (MPa)-DFT		*σ*_b_ (MPa)-FT	
		Para.	Perp.	Para.	Para.
10.0	2.0	1.05	0.72	0.95	0.90
10.0	4.0	1.42	0.90	1.45	1.38
10.0	8.0	1.85	1.15	1.72	1.76
10.0	0	0.55	0.40	0.58	0.52

**Fig. 4:**
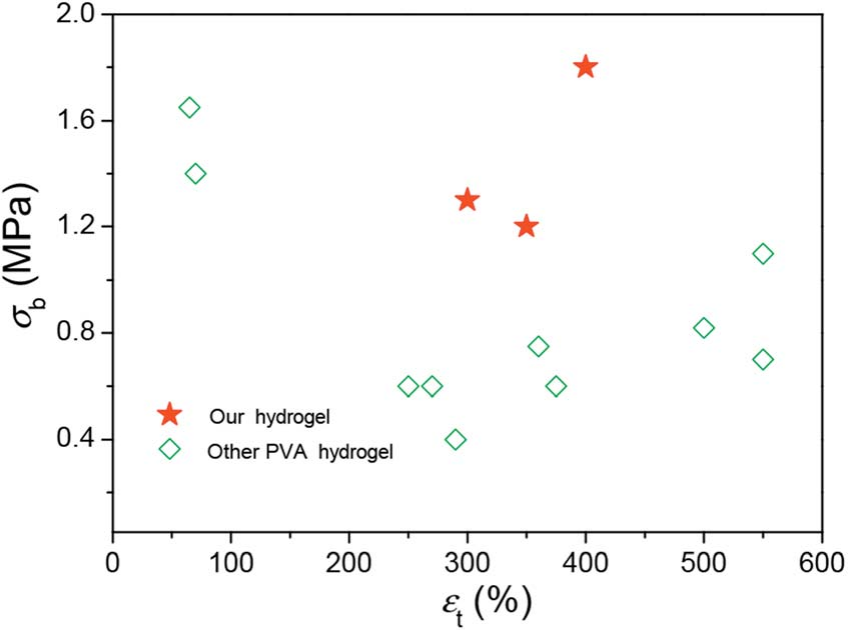
A summary of the fracture strengths and elongations of hydrogels with high mechanical strengths.

These results demonstrated that the DFT PVA/GO hydrogel exhibit good mechanical properties in the presence of GO, which can be attributed to its excellent original mechanical strength and the formation of physical cross-links interactions between graphene sheets and polymer chains. The breaking strength of our gels is higher than other PVA gels at containing water of 90% (Fig. 4),^14,24,25^ it might provide a possible for fabricating some artificial soft tissues.

**Fig. 5:**
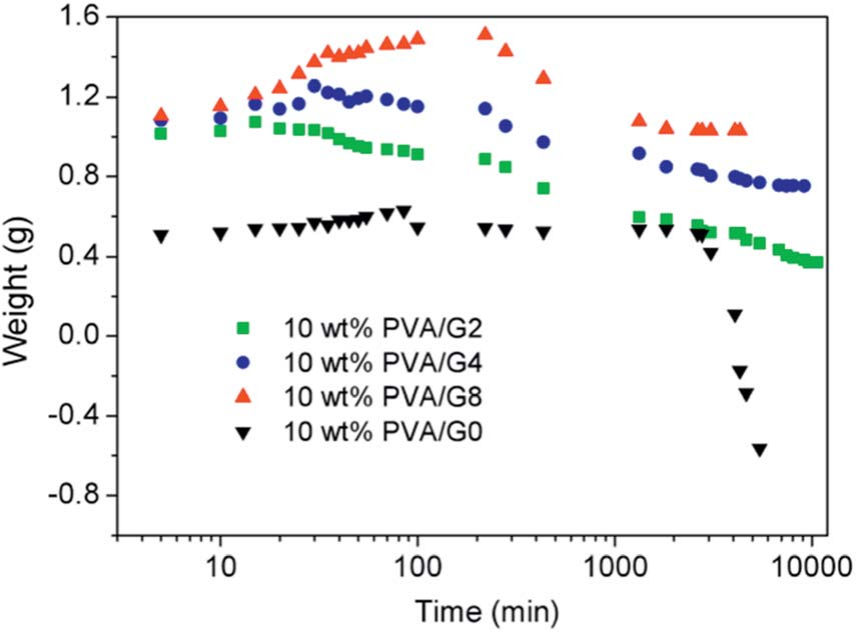
Swelling curves of PVA/GO hydrogels at 65 °C.

**Fig. 6:**
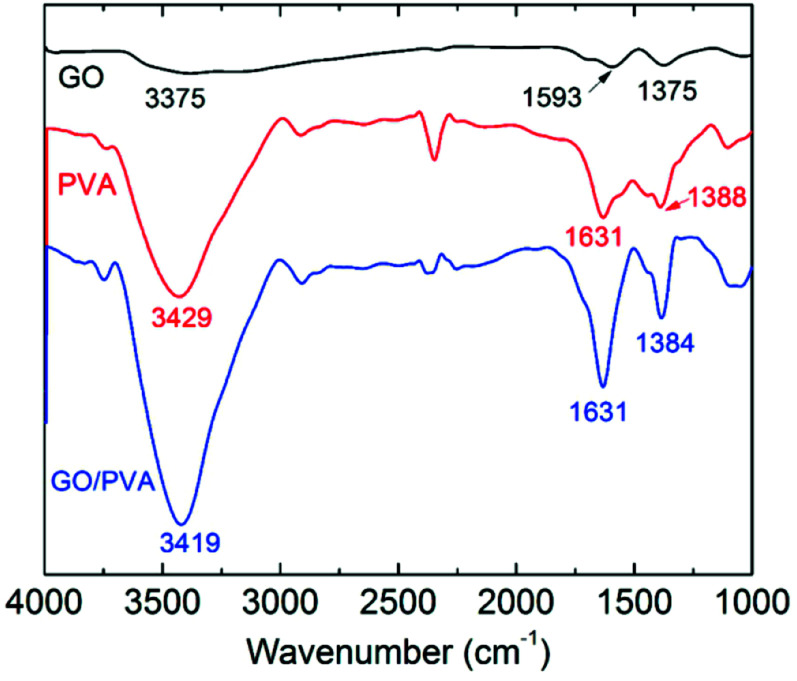
FTIR spectra of dried GO, PVA, and PVA/GO composites hydrogels.

The thermostability properties of the nanocomposites hydrogel are further improved in the presence of the more oxygen groups among GO and PVA molecules. Based on this, we further investigated the swelling behavior of hydrogels at high temperature. Firstly, four gel samples with different *C*_GO_ were swollen in the hot water (65 °C), and the changes of sample mass were recorded with the increasing of swelling time. As shown in Fig. 5, at the beginning of the swelling, the sample mass showed a slow rising trend due to absorbing more water to reach to the swelling equilibrium. In this experiment, it is worth to notice that the sample needs more time to achieve the swelling equilibrium with the increasing of GO concentration, which is mostly attributed to improvement of hydrophily after introduction of more oxygen-containing groups. For example, the equilibrium swelling water contents of PVA/GO8 was 97% after swollen treatment for 220 min, which was much longer than the other samples. After that, the gel samples began to be dissolved with the increasing of swelling time, but the weight of PVA/GO8 gel down to about 1.0 g and then remained almost constant, while PVA/GO0 gel was almost dissolved totally. The result indicated that the interaction between PVA chains and graphene sheets^26,27^ can be beneficial to the improvement of thermostability for PVA/GO gels, mainly attributed to strong intermolecular hydrogen bonds with PVA molecules (hydroxyl groups) and GO sheets. A reliable evidence for the formation of hydrogen bonding in the PVA/GO gel was revealed from FTIR characterizations of GO, PVA, and PVA/GO hydrogels. As shown in Fig. 6, FTIR spectrum of GO shows the typical absorption bands attributed to hydroxyl (–OH), carbonyl (C=O) and carboxy (C–O) groups at 3375, 1593 and 1375 cm^−1^ stretches,^20^ while the bands for pure PVA and the PVA/GO samples shifted to 3429, 1631, 1388 cm^−1^, and 3419, 6131, 1384 cm^−1^, respectively, suggesting the formation of hydrogen bonding between GO sheets and PVA chains.

**Fig. 7:**
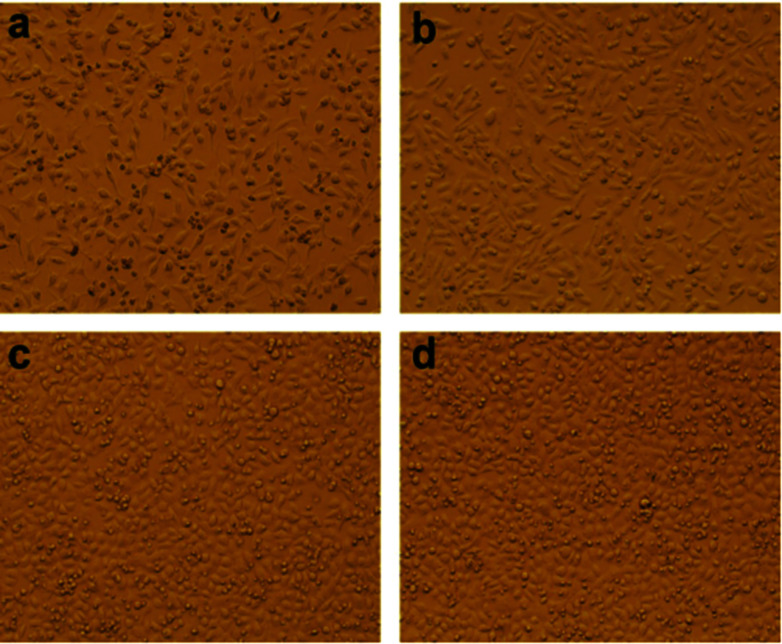
HeLa cells cultured on PVA/GO2 composite hydrogels after inoculation for 1 (a), 3 (b), 5 (c) and 7 (d) days.

The excellent biocompatibility is the basic requirement for hydrogel biomaterials in clinical application.^28^ We carried on the preliminary evaluation for the biocompatibility of PVA/GO composite hydrogels by a cell culture experiment. In this study, we chose the HeLa as the passage cells. Specifically, the gel samples were soaked in the culture medium for 24 h, and then a certain amount of HeLa cells were adhered to the gel samples as substrate to carry on an adherent culture experiment in the culture medium. As shown in Fig. 7, the morphological changes in HeLa cells cultured on PVA/GO composite hydrogels were observed under the microscope after growing for 1 (a), 3 (b), 5 (c) and 7 (d) days. With the increasing of the culture time, the pebbles-like HeLa cells were adherent and growing fast observably. Although the number of dead cells also increased slowly, most of the cells also showed the aggregation growth in the field of vision, suggesting that the cells grow well in the culture medium. The above results demonstrated that PVA/GO composite hydrogels exhibit the excellent biocompatibility, and it may provide the possibility for the further study in biomedical applications. Drug-releasing is one of the important applications in the field of hydrogel materials. PVA hydrogel, as a drug release carrier, is the optimal choice because of its good hydrophilicity and biocompatibility. In this work, VB12 was used as drug models to preliminary investigate the controlled drug release effect of the PVA/GO hydrogels.

**Fig. 8:**
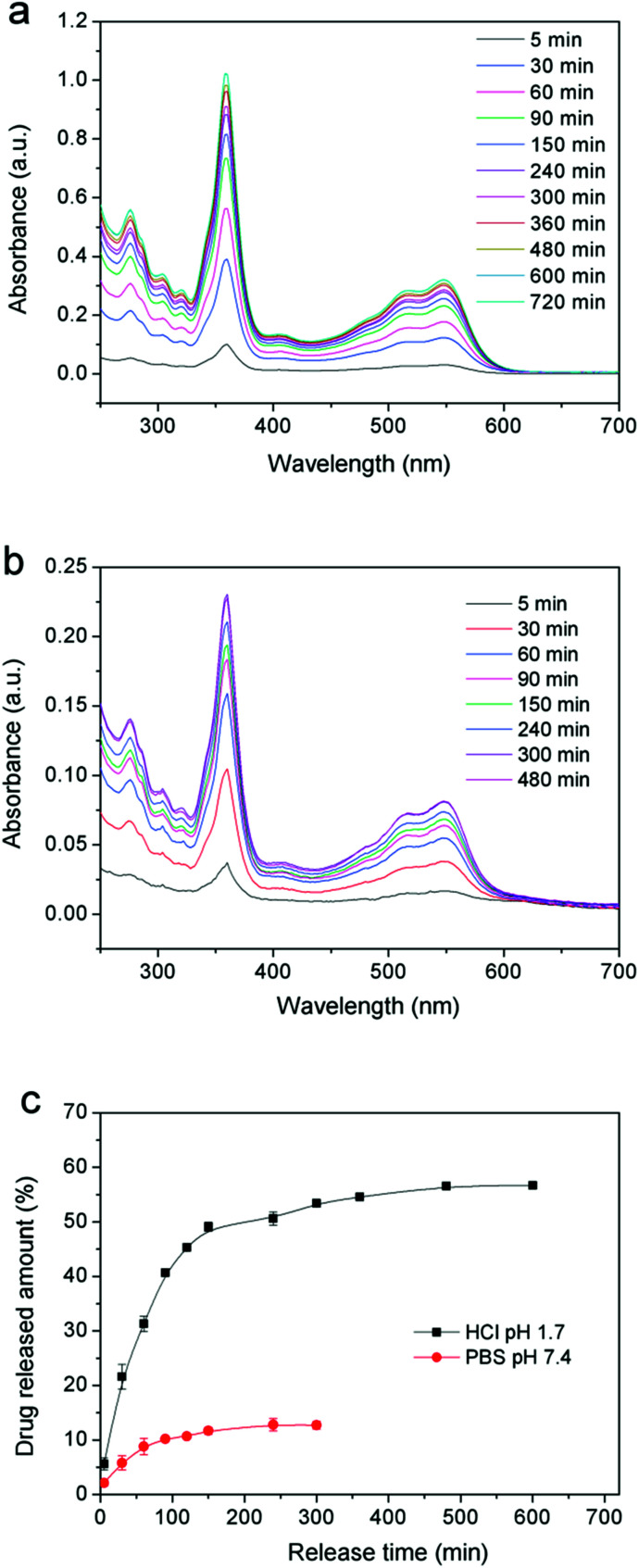
UV-visible spectra of VB12 released in (a) HCl (pH = 1.7), (b) PBS (pH = 7.4) solutions and (c) profiles of releasing VB12 from a PVA/G4 composite hydrogel in two solutions.

Fig. 8a and b show that the ultraviolet absorption curves of VB12 in two kinds of pH environment at different times. With the increase of release time, the absorption intensity was increasing gradually, indicating that the amounts of drug release in the gel system were also increasing. Specifically, in dilute HCl solution, the amount of drug release increased rapidly within 90 min and the VB12 release rate reached about 40% (Fig. 8c), after then the release became more slowly and only a slight increase appears between 200 and 700 min. Finally, the total amounts of drug release were up to 55% after 12 h. Compared with HCl solution, the drug release ability of the resulting hydrogels decreased significantly after swelling in PBS buffer solution. In this experiment, it should be noted that the drug release rate did not change too much and maintain in the amount about 12% after 8 h later releasing. These results clearly indicated that PVA/GO composite gels could play an important role in drug carriers, and exhibit the desired controllability of drugs release in the environment of simulated gastric juice.

The ideal drug controlled-release system should provide the drug supply keeping slow and steady, which can be contributed to play efficacy adequately, so that it could greatly avoid the side effect of drug to human's healthy. Utilizing the PVA/GO composite gels pH-sensitive, we realized the controlled release to VB12. Generally speaking, at low pH (1.7), the layer size of GO in composite hydrogels would be affected, which is mainly attributed to electrostatic repulsion between GO sheets.^29^ In this case, the loaded drug (such as VB12) may also be influenced by the change of graphene sheets. And the large size of graphene provides the inner driving force for the controlled-release system and it makes the drug molecules moving to low concentration area after the structure of graphene sheets changed. Meanwhile, the directional freezing technology gives the oriented microstructures to our drug release system, which could provide more micro-channels for drugs, so that it could be released more easily and steadily finally. When in a neutral environment, the gels internal structures do not change too much, and the releasing of small amount of drug dissociated in the gel system is mainly due to the pressure difference between inside and outside during the swelling process.

## Conclusions

In summary, we demonstrated the fabrication of a tough PVA/GO nanocomposite hydrogels with anisotropic microstructure and mechanical properties through a facile and effective directional freezing–thawing method. The resulting gels show anisotropic lamellae microstructures in the direction parallel to the freezing direction and porous in the perpendicular direction. The PVA/GO composite hydrogels with high water contents (90 wt%) exhibit higher fracture strength (up to 1.85 MPa) in the parallel direction with the aid of GO nanosheets and DFT process. The anisotropic mechanical properties of the PVA/GO gels have been dramatically enhanced with the increasing of *C*_GO_. Moreover, the applications of drug released and biocompatibility were investigated. The hydrogel materials may expand the more practical applications in biomedical and engineering fields, such as scaffold for biomimetic bone, soft robots, *etc.*

## Conflicts of interest

The authors declare no competing financial interest.

## Supplementary Material
